# Investigating the effect of corneal Herpes Simplex Virus-1 infection on Toll-Like Receptor expression in human peripheral blood mononuclear cells

**DOI:** 10.1186/1753-6561-6-S4-P38

**Published:** 2012-07-09

**Authors:** T Chan, B Coffey, D Shahnazaryan, C Jefferies, C Murphy

**Affiliations:** 1 Royal College of Surgeons in Ireland, Ireland

## Introduction

Herpes Simplex keratitis (HSK), caused by Herpes Simplex Virus type 1 (HSV-1), is the leading cause of infectious corneal blindness in the western world [1]. Initial infection develops through ocular surface entry from droplet spread [2]. Once HSV-1 has breached the epithelial barrier of the ocular surface, it is recognized by Toll-Like Receptors (TLRs), which then activate the appropriate innate immune response. Despite the high prevalence of HSV-1, only a small minority of patients develop ocular manifestations. Therefore, we hypothesized that TLR expression and activity may be deregulated in patients with HSK, which would reflect in peripheral blood mononuclear cell (PBMC) responses observed in these patients. We investigated the effects of the TLR ligands 3, 4, 7 and 9 (as they are involved in anti-viral immune defence [3]) on cytokine induction from a patient with active HSK and compared responses of this patient to TLR ligands on a subsequent follow up visit where disease was diagnosed to be inactive.

## Methods

PBMCs were isolated with Ficoll-Paque Plus density gradient centrifugation. Cells were stimulated with ligands to TLRs 3, 4, 7 and 9 - polyinosinic: polycytidylic acid (poly I:C), lipopolysaccharide (LPS), imiquimod (IMIQ), and CpG A, B, and C, respectively, for 4hrs and overnight. Following stimulation, supernatant was removed for measurements of TNF-α and IL-6, which were determined by enzyme-linked immunosorbant assay (ELISA). mRNA was extracted from stimulated cells using the Trizol method and changes in levels of TLR expression were quantified by qPCR.

## Results

The overall qPCR result showed that during the active phase of HSK the patient analysed in this study had higher TLR expression than when disease had resolved. In the active patient, TLR 4 expression was particularly high when stimulated with poly I:C, IMIQ and CpG C. TLR 3 and 7 showed a moderate increase when stimulated with LPS and poly I:C, whereas TLR 9 expression remained low throughout. Differences in IL-6 and TNF-α production were also observed when samples similarly stimulated were analysed.

## Conclusions

In the active patient, the increased TLR expression correlates with the increased levels of cytokines TNF-α and IL-6 in the ELISAs. This means that the entire TLR signalling pathway is functioning at a higher level when the patient is actively infected with HSV-1.
